# Detection of Second Line Drug Resistance among Drug Resistant Mycobacterium Tuberculosis Isolates in Botswana

**DOI:** 10.3390/pathogens8040208

**Published:** 2019-10-28

**Authors:** Tuelo Mogashoa, Pinkie Melamu, Brigitta Derendinger, Serej D. Ley, Elizabeth M. Streicher, Thato Iketleng, Lucy Mupfumi, Margaret Mokomane, Botshelo Kgwaadira, Goabaone Rankgoane-Pono, Thusoyaone T. Tsholofelo, Ishmael Kasvosve, Sikhulile Moyo, Robin M. Warren, Simani Gaseitsiwe

**Affiliations:** 1 Department of Medical Laboratory Sciences, Faculty of Health Sciences, University of Botswana, Gaborone 0022, Botswana; tuelomogashoa@me.com (T.M.); lmupfumi@gmail.com (L.M.); kasvosvei@ub.ac.bw (I.K.); 2 Botswana Harvard AIDS Institute Partnership, Gaborone 0000, Botswana; pmpmelamu@gmail.com (P.M.); iketlengt@gmail.com (T.I.); sikhulilemoyo@gmail.com (S.M.); 3 DST-NRF Centre of Excellence in Biomedical Tuberculosis Research, South African Medical Research Council Centre for Tuberculosis Research, Division of Molecular Biology and Human Genetics, Faculty of Medicine and Health Sciences, Stellenbosch University, Tygerberg, Cape Town 7505, South Africa; brigitta@sun.ac.za (B.D.); 21280606@sun.ac.za (S.D.L.); lizma@sun.ac.za (E.M.S.); rw1@sun.ac.za (R.M.W.); 4 Department of Medical Parasitology and Infection Biology, Swiss Tropical and Public Health Institute, Basel 4002, Switzerland; 5 Faculty of Science, University of Basel, Basel 4001, Switzerland; 6 College of Health Sciences, School of Laboratory Medicine and Medical Sciences, University of KwaZulu-Natal, Durban 4041, South Africa; 7 National Tuberculosis Reference Laboratory, Ministry of Health and Wellness, Gaborone 0000, Botswana; bafanamargaret@gmail.com; 8 Botswana National Tuberculosis Programme, Ministry of Health and Wellness, Gaborone 0000, Botswana; btkgwaadira@gmail.com (B.K.); goaba2000@yahoo.com (G.R.-P.); ttsholo808@gmail.com (T.T.T.); 9 Department of immunology and infectious diseases, Harvard T.H. Chan School of Public Health, Boston, Massachusetts, MA02115, USA

**Keywords:** *Mycobacterium tuberculosis*, line probe assay, second-line drugs, drug resistance, XDR-TB, MDR-TB

## Abstract

The emergence and transmission of multidrug resistant (MDR) and extensively drug resistant (XDR) *Mycobacterium tuberculosis (M.tb)* strains is a threat to global tuberculosis (TB) control. The early detection of drug resistance is critical for patient management. The aim of this study was to determine the proportion of isolates with additional second-line resistance among rifampicin and isoniazid resistant and MDR-TB isolates. A total of 66 *M.tb* isolates received at the National Tuberculosis Reference Laboratory between March 2012 and October 2013 with resistance to isoniazid, rifampicin or both were analyzed in this study. The genotypes of the *M.tb* isolates were determined by spoligotyping and second-line drug susceptibility testing was done using the Hain Genotype MTBDR*sl* line probe assay version 2.0. The treatment outcomes were defined according to the Botswana national and World Health Organization (WHO) guidelines. Of the 57 isolates analyzed, 33 (58%) were MDR-TB, 4 (7%) were additionally resistant to flouroquinolones and 3 (5%) were resistant to both fluoroquinolones and second-line injectable drugs. The most common fluoroquinolone resistance-conferring mutation detected was *gyrA* A90V. All XDR-TB cases remained smear or culture positive throughout the treatment. Our study findings indicate the importance of monitoring drug resistant TB cases to ensure rapid detection of second-line drug resistance.

## 1. Background

In 2017, 10 million people fell ill with tuberculosis (TB) and 1.6 million people died of TB [1]. In the same year, an increase in cases of rifampicin monoresistant and multidrug resistant TB (MDR-TB defined as TB that is resistant to rifampicin and isoniazid) from 490,000 in 2016 to 558,000 in 2017 was observed [1]. The increasing numbers of rifampicin and MDR-TB cases poses a risk to TB control programs throughout the world [2]. Rifampicin monoresistance is considered to be a precursor to MDR-TB and there are often concerns about rifampicin monoresistant TB patients acquiring MDR-TB [3,4]. The standardized World Health Organization (WHO) MDR-TB treatment regimen recommends the use of second-line injectable drugs (SLIDs) in combination with flouroquinolones as part of the standardized MDR-TB treatment regimen [2]. The resistance to a fluoroquinolone and a SLID negatively impacts treatment outcome and has been defined as extensively drug resistant TB (XDR-TB) [5,6,7]. MDR-TB in combination with resistance to either a fluoroquinolone or a SLID has been termed Pre-XDR-TB. 

The resistance to fluoroquinolones is usually caused by point mutations in the quinolone resistance determining region (QRDR) of the gene encoding subunit A or B of the deoxyribonucleic acid (DNA) gyrase gene (*gyrA* or *gyrB*) [8,9,10]. In the *gyrA* gene, the resistance mutations are commonly found in codons 85 to 96, whereas for the *gyrB* gene, they are found in codons 472 and 510 [5,6]. The mutations between codon 1400 and 1500 in the *rrs* gene are often associated with resistance to SLIDs such as capreomycin, kanamycin and amikacin [5]. The timely detection of resistance to SLIDs remains critical for optimizing treatment to improve the treatment outcome as well as directing infection control measures to halt the transmission of drug resistant TB [7,11]. Diagnostic assays, such as the Hain GenoType MTBDR*sl* line probe assay (Hain Lifescience, Germany), have been endorsed by WHO for the rapid detection of second-line drug resistance [11]. The MTBDR*sl* test is based on the DNA strip technology which has three steps: DNA extraction, multiplex polymerase chain reaction (PCR) amplification and reverse hybridization [12]. This assay has proven to be reliable for rapidly detecting resistance to second-line drugs [13] and has been implemented in 28 countries in Africa [14]. Monitoring drug resistance with the help of such assays and evaluating treatment outcomes may help improve management of TB [15,16].

This study sought to determine the level of resistance to second-line drugs among rifampicin monoresistant, isoniazid monoresistant and multi-drug resistant TB cases in Botswana using the Hain genotype MTBDR*sl* Version 2.0 and to assess patient treatment outcomes. 

## 2. Methods

### 2.1. Design and Study Population 

This was a retrospective, cross-sectional study utilizing *M.tb* isolates from the Botswana National Tuberculosis Reference Laboratory (NTRL) bio-repository which were collected as part of routine clinical care between 2012 and 2013. The study was approved by the University of Botswana Ethics Institutional Review Board and Health Research and Development Committee (HRDC) at the Ministry of Health and Wellness (Reference No: HPDME: 13/18/1 Vol. XI (140)). Sixty-six *M.tb* isolates were selected. The selected isolates were isoniazid (H) or rifampicin (R) monoresistant or resistant to both (MDR) based on first-line culture-based drug susceptibility testing (DST). The isolates included in this study are part of a previously described larger study and culture-based drug susceptibility testing for first-line drugs was done as previously described [17]. The clinical treatment outcome data was obtained from the Botswana National Tuberculosis Program (BNTP) patient database. At the time of the study, the standardized MDR-TB treatment regimen in Botswana consisted of pyrazinamide, amikacin, levofloxacin, ethionamide, cycloserine, P-aminosalicylic acid (PAS) [18].

### 2.2. Treatment Outcome Definitions

Treatment outcomes were defined according to the Botswana national guidelines. Briefly, “cured” was defined as a patient whose smear or culture sample was positive at the start of treatment but either converted to smear negative or had two consecutive negative cultures, one during treatment and the other at the end of treatment; “failed” was defined as a patient whose smear or culture was positive five months or later during treatment; “loss-to-follow-up” was defined as a patient whose treatment was interrupted for more than 30 consecutive days; “not evaluated” referred to patients whose treatment outcome could not be assigned since treatment conclusion has not been reported to the national TB program; treatment “completed” referred to patients who completed treatment but did not have a negative smear or culture result in the last month of treatment [18]. In bivariate comparisons, treatment outcomes were combined: “failed treatment” (i.e. remaining smear or culture positive throughout treatment), “loss-to-follow-up”, death, “not initiated on treatment” and “not evaluated” as “unsuccessful treatment outcome” and “completed treatment”, “cured” as “successful treatment outcomes”.

### 2.3. DNA Extraction

DNA was extracted from the BD MGIT960 cultures (BD Biosciences, Sparks, MD, USA) using the GenoLyse DNA extraction kit version 1.0 (Hain LifeScience, GmBH, Nehren, Germany) following the manufacturer’s instructions [19].

### 2.4. Genotyping

#### 2.4.1. Spoligotyping

The genotypes of the isolates were determined by spoligotyping as previously described by Kamerbeek et al. [20] and Mogashoa et al. [17]. The *M.tb* families and lineages of the isolates were assigned based on the spoligotyping results.

#### 2.4.2. Hain Genotype MTBDRsl Version 2

The second line drug resistance profiles were determined by using the Hain GenoType MTBDR*sl* assay (Hain Lifescience, Germany). The steps were performed as per the manufacturer’s instructions [12]. The culture based second line phenotypic DST was not performed for this study since the test was unavailable at the reference laboratory.

#### 2.4.3. Data Analysis

Fischer’s exact test was used to determine if there was an association between second line drug resistance and *M.tb* family, the patients’ age, HIV status and sex. The factors were examined for a favorable treatment outcome using logistic regression techniques. A *p*-value of <0.05 was considered statistically significant. STATA version 14 (Stata Corp, College Station, TX, USA) was used for statistical analysis.

## 3. Results

A total of 57 out of 66 (86%) isolates (one isolate per patient) were successfully genotyped and tested for resistance to first-line drugs (culture-based phenotypic DST) and second-line drugs (line probe assay MTBDR*sl*). Of these 57 isolates, 27 (47.4%) were from the southern region, 24 (42.1%) were from the central region, 5 (8.8%) from north west and 1 (1.8%) from south west region. The median age of the patients was 34 years [Q_1_, Q_3_: 13,59] with half (50%) being in the 20–39 years age group. For those patients with a known HIV status, 31 (54.4%) were HIV positive, 15 (26.3%) were HIV negative and 11(19.3%) had an unknown HIV status. The *M.tb* lineages identified among the DR-TB isolates were Lineage 4 (66.7%), Lineage 2 (19.3%), Lineage 1 (12.3%) and unknown lineage (1.8%) (Table 1). 

**Figure 1:**
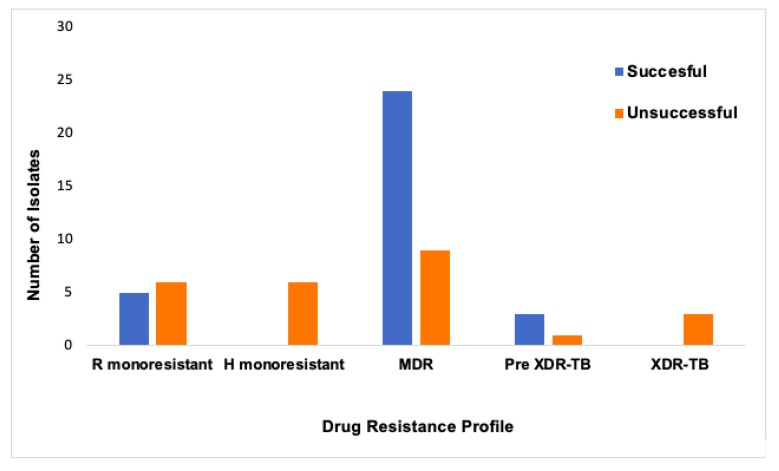
Treatment outcomes of patients with different drug resistance profiles.

Among the 57 drug resistant isolates, the first and second-line DST results showed that 19% of the cases were resistant to rifampicin only, 11% were resistant to isoniazid only, 58% were resistant to both isoniazid and rifampicin (MDR), 7% of the MDR isolates showed additional resistance to flouroquinolones (pre-XDR) while 5% of the MDR isolates were resistant to flouroquinolones and SLIDS (XDR). This study did not find any pre-XDR isolates with SLID resistance. The treatment was successful in 75% of the pre-XDR-TB cases, whereas all XDR-TB cases had unsuccessful treatment outcomes. All isoniazid monoresistant cases had unsuccessful treatment outcomes; 55% of the rifampicin monoresistant cases had unsuccessful treatment outcomes; among the MDR-TB cases, 73% had successful treatment outcomes (Figure 1). No statistically significant association was found between the second line drug resistance or the treatment outcome with HIV status, age, sex and *M.tb* family (Table 2). Table 3 shows characteristics and treatment outcomes of pre-XDR and XDR-TB cases in the study. The treatment outcomes for the rest of the cases are shown in supplementary Table S1. When evaluating the MTBDR*sl* results, it was found that the most common fluoroquinolone-resistance conferring mutation detected was *gyrA* A90V (found in 7% of the cases). The mutation *gyrA* G88A/G88C was only detected in one isolate. Among the pre-XDR-TB and XDR-TB cases, the second line injectable drug resistance was caused by the mutation *rrs* A1401G. Of the 7 pre-XDR and XDR-TB patients, the HIV status was not known for two patients, while the other five patients were HIV positive. Some patients with known HIV status had the same hybridization pattern, drug resistance profile, *M.tb* lineage and spoligo family as patients with unknown HIV status (Table 3). 

R- Rifampicin; H- isoniazid; MDR-TB- Multi-drug resistant TB; Pre-XDR-TB- Pre-extensively drug resistant tuberculosis; XDR-TB-extensively drug resistant tuberculosis. “Successful treatment”; includes patients who completed treatment and those who were cured; “Unsuccessful treatment”; includes patients who failed treatment, patients who are deceased, loss to follow up, defaulted, not evaluated and not initiated into treatment. 

## 4. Discussion

In this retrospective analysis of drug resistant isolates from Botswana, it is shown that there is a high proportion of rifampicin (R) and isoniazid (H) monoresistance posing an increased risk of the development of MDR-TB [21,22]. It was observed that 7% (4/57) of the isolates were pre-XDR-TB. These isolates had resistance to fluoroquinolone only while 5% (3/57) were XDR-TB. The majority, 88% (50/57) of the isolates did not have any resistance to second-line drugs. It is interesting to see that in this sample, set SLID resistance occurred after fluoroquinolone resistance and not the other way around. Fluoroquinolones are used to treat other bacterial infections other than TB which could play a role in the increasing levels of resistance to this class of drugs in *M.tb* (in both pre-XDR and XDR cases) [5]. The introduction of Pretomanid, recently approved by the Food and Drug Administration (FDA) for the treatment of R resistant TB, has greatly shortened treatment and has been seen to improve treatment success [23,24]. However, Pretomanid is not yet available in Botswana. The presence of both pre-XDR-TB and XDR-TB cases are indicators that there are gaps in the control of TB. The genotyping methods used in this study are not sufficiently discriminatory to investigate transmission, and whole genome sequencing has not been done on these strains. However, the possibility exists that patient to patient transmission exists as there were 2 patients who were infected with a strain of the same spoligotype and second-line drug resistance pattern. Further analysis would however be needed to investigate TB transmission in this population. 

The GenoType MTBDR*sl* assay can detect mutations in the quinolone resistance determining region (QRDR) of the genes *gyrA* and *gyrB*. The most common *gyrA* mutation detected by the MTBDR*sl* among the pre-XDR and XDR cases was A90V (57%). This mutation confers resistance to levofloxacin and is associated with low level resistance to moxifloxacin [8]. Some resistance mutations are characterized by the absence of hybridization at the respective wild type probes [25]. The absence of the wild type bands in the line probe assay can be used to infer that there could be resistance to flouroquinolones but it does not allow the determination of the genotypic changes and the resulting phenotypic resistance to specific drugs. The targeted sequencing is therefore required to identify the specific drug resistance mutations. For example, in our cohort, there were two isolates which had an undefined mutation shown by the absence of both the wild type (*gyrA* WT3) and the mutation band in the *gyrA* gene. In this case, resistance to fluoroquinolones, particularly levofloxacin, can only be inferred since the specific mutation is not known [25]. This information can nevertheless help select a treatment regimen that could be more beneficial to the patients (e.g., excluding fluoroquinolones). The future implementation of new drugs, such as Bedaquiline and Pretomanid, could change the genetic drug resistance patterns observed in this study. The pre-XDR and XDR-TB patients with drug resistance patterns described in this study could still be successfully treated with these drugs, thereby reducing the spread of these *M.tb* strains. The genotypic drug resistance patterns therefore need to be closely monitored to be able to adapt treatment guidelines if required. 

This study found that all the XDR-TB patients had unsuccessful treatment outcomes. These strains being XDR probably resulted in (almost) none of the prescribed drugs being efficient in killing the bacteria. The pre-XDR and XDR-TB patients in this study were managed with regimens which contained levofloxacin. Previous studies have shown that in cases of levofloxacin resistance, moxifloxacin may be the preferred drug of choice since *gyrA* A90V mutation has a smaller effect on moxifloxacin activity [8,26]. Therefore, these patients could have benefited from a regimen containing Moxifloxacin if the specific resistance markers had been determined timely. Among the pre-XDR isolates and MDR-TB isolates, 75% and 73% of the patients had successful treatment outcomes respectively, however all isoniazid monoresistant and 55% of the rifampicin monoresistant patients had unsuccessful treatment outcomes. Previous studies have shown that isoniazid monoresistance is associated with poor treatment outcomes [3,27].

There were no mutations detected in the *gyrB* gene in any of the isolates in this study. The mutations in the *gyrB* gene are usually associated with low level resistance to fluoroquinolones and are not as common as those in the *gyrA* gene [2]. The *rrs* MUT1 A1401G mutation which leads to a high level second-line injectable drug resistance was detected among 5% of the MDR-TB isolates. This mutation causes high level resistance to KAN and cross resistance to AM and CAP [6]. The presence of these mutations shows that there is a need to routinely test for second-line drug resistance among MDR-TB cases in Botswana. In this study, gene mutations that are associated with low level drug resistance induced by mutations in the promoter area of the *eis* gene were not detected. There was no association between the drug resistance profile and HIV status. The data on HIV viral load and CD4 cell counts were not available for this study and their association with drug resistance could therefore not be analyzed. However, previous studies have shown that there is an association between drug resistance and HIV status [28,29,30]. Haar et al. and Fenner et al. have shown that patients with high viral loads are more likely to have multi-drug resistant TB than those who are virally suppressed [31,32]. 

Even though this study is informative and provides data on the genetic mutations that are associated with second-line drug resistance in Botswana, it had some limitations. The small sample size may not reflect the true burden of second-line drug resistance in the entire country and there is limited statistical power to detect other drug resistance mutations within the population. Due to the small sample size, there is insufficient statistical power to fully address the association with various risk factors and treatment outcomes. This was a retrospective study therefore, there may be other unknown confounding factors. One of the limitations of the line probe assays (LPAs) is that there may be a false detection of resistance due to some synonymous mutations. Some studies have reported synonymous mutations which can result in false-positive results (false detection of resistance). However, in such instances, appropriate confirmatory testing should be done promptly [33]. The lack of hybridization of the wild type probes is not a reliable indication of phenotypic resistance, hence these kinds of hybridization patterns need to be verified with phenotypic DST [7]. There were some mutations that were undefined in our study therefore, other mechanisms of fluoroquinolone resistance need to be investigated further using techniques, such as whole genome sequencing or targeted gene sequencing. The identification of gyrase mutations can aid in predicting fluoroquinolone resistance as well as estimating the levels of resistance to various flouroquinolones. This may assist clinicians to determine the most effective dose of fluoroquinolones [34]. There is also a need to carry out this study in a larger population in order to determine the association of several risk factors with treatment outcomes as well as to determine the prevalence of second-line drug resistance in Botswana. 

## 5. Conclusions

Our study shows that there is second-line drug resistance and the majority of cases had *gyrA* A90V, *rrs* A1401G mutation. Our results show that monitoring and further investigations with more discriminatory methods are required to determine whether these strains are transmitted or if second-line drug resistance is acquired during treatment.

## Figures and Tables

**Table 1:** Demographic characteristics of patients included in the study (n = 57).

	n	%
Sex		
Male	29	50.9
Female	28	49.1
Age in years		
<20 years	9	16.1
20–39 years	28	50.0
40–59 years	16	28.6
>60 years	3	5.4
HIV status		
Negative	15	26.3
Positive	31	54.4
Unknown	11	19.3
Specimen type		
Extra-pulmonary	1	1.8
Pulmonary	55	96.5
Unknown	1	1.8
Smear results		
Negative	12	21.1
Positive	45	79
Drug resistance profile		
Rifampicin monoresistant	11	19.3
Isoniazid monoresistant	6	10.5
Multi-drug resistant (MDR)	33	57.9
Pre-XDR*	4	7.0
XDR**	3	5.3
Region		
Central	24	42.1
South West	1	1.8
North West	5	8.8
Southern	27	47.4
Lineage		
Lineage 1	7	12.3
Lineage 2	11	19.3
Lineage 4	38	66.7
Unknown	1	1.8

*Pre-XDR: Pre-extensively drug resistant. **XDR: extensively drug resistant.

**Table 2:** Factors associated with second line drug resistance.

	MDR N = 50	2^nd^ Line Drug Resistance* N = 7	*p*-value
Sex	n (%)	n (%)	0.253
Male	27 (54)	2 (29)	
Female	23 (46)	5 (71)	
Age in years			0.833
<20 years	8 (16)	1 (14)	
20–39 years	23 (47)	5 (71)	
40–59 years	15 (31)	1 (14)	
>60 years	3 (6)	0 (0)	
HIV status			0.226
Negative	15 (30)	0 (0)	
Positive	26 (52)	5 (71)	
Unknown	9 (18)	2 (29)	
Smear results			0.630
Negative	10 (20)	2 (29)	
Positive	40 (80)	5 (71)	
Region			0.866
Central	20 (40)	4 (57)	
South West	1 (2)	0 (0)	
North West	24 (48)	0 (0)	
Southern	5 (10)	3 (43)	
Lineage			0.066
Lineage 1	4 (8)	3 (43)	
Lineage 2	11 (22)	0 (0)	
Lineage 4	34 (68)	4 (57)	
Unknown	1 (2)	0 (0)	

*Second-line drug resistance includes pre-XDR and XDR-TB patients.

**Table 3:** Characteristics of XDR-TB and pre-XDR-TB cases detected among the drug resistant cases.

Case	Age	Sex	Region	HIV Status	FLDs Drug Resistance Pattern	Hybridization Pattern (s)	Codon Mutations	SLDs Drug Resistance Pattern	*M.tb* Lineage, Spoligo Family	Treatment Outcome
**1**	29	F	Central	Positive	H; R; S; E	*gyrA* ΔWT3 *+ rrs* ΔWT1 + *rrs* MUT1	Undefined mutation, A1401G	OFL; LFX; KAN; AM; CAP	L4, X3	Failed
**2**	28	F	North West	Unknown	H; R; S; E	*gyrA* ΔWT3 *+ rrs* ΔWT1 + *rrs* MUT1	Undefined mutation, A1401G	OFL; LFX; KAN; AM; CAP	L4, X3	Failed
**3**	32	M	North West	Positive	H; R; E	*gyrA* ΔWT2 *+ gyrA MUT1+ rrs* ΔWT1 + *rrs* MUT1	A90V, A1401G	OFL; LFX; KAN; AM; CAP	L4, LAM4	Deceased
**4**	37	F	Central	Positive	H; R; E	*gyrA* ΔWT3 + *gyr*A MUT1	A90V	OFL; LFX	L1, EAI1_SOM	Completed
**5**	44	M	Central	Unknown	H; R; S; E	*gyrA* ΔWT2 + *gyrA* MUT1	A90V	OFL; LFX	L1, EAI1_SOM	*Not evaluated
**6**	34	F	Central	Positive	H; R; S; E	*gyrA* ΔWT2 + *gyrA* MUT1	A90V	OFL; LFX	L1, EAI1_SOM	Completed
**7**	16	F	South	Positive	H; R; S; E	*gyrA* ΔWT1	G88A/G88C	OFL; LFX	L4, LAM3	Cured

H: Isoniazid; R: Rifampicin; S: Streptomycin; E: Ethambutol; FLDs: first-line drugs; SLDs: second-line drugs; OFL: Ofloxacin; LFX: Levofloxacin; KAN: Kanamycin; AMK: Amikacin; CAP: Capreomycin: L4: Lineage 4; L1: Lineage 1. *Patient was not initiated on treatment. WT- wild type; ΔWT1, ΔWT2, ΔWT3- no hybridization at respective WT probe; MUT1-mutation.

## Supplementary Materials

The following are available online at https://www.mdpi.com/2076-0817/8/4/208/s1, Table S1. Clinical characteristics and treatment outcomes of the drug resistant isolates in the study.
