# Supplementary material for: Detection of Second Line Drug Resistance among Drug Resistant Mycobacterium Tuberculosis Isolates in Botswana
Source: Pathogens. 2019 Oct 28;8(4):208. doi: 10.3390/pathogens8040208 (PMC6963291; doi:10.3390/pathogens8040208)
Supplement: Supplementary file 1 [file pathogens-08-00208-s001.pdf]

pathogens

MDPI

Table S1. Clinical characteristics and treatment outcomes of the drug resistant isolates in the study.

Case	Age (years)	Sex	Region	HIV Status	FLDs Resistance Pattern	SLD Resistance Pattern	*M.tb* Lineage; Spoligo Family	Treatment Outcome
**1**	24	F	North	Negative	H; R; E	Susceptible	L2; Beijing	Completed
**2**	35	M	North	Negative	H; R; E; S	Susceptible	L2; Beijing	Completed
**3**	43	F	South	Positive	H; R;	Susceptible	L4; S	LTFU
**4**	24	F	South	Negative	H; R; E; S	Susceptible	L4; LAM3	Completed
**5**	17	F	South	Negative	H; R	Susceptible	L4; X3	Cured
**6**	Unknown	F	South	Positive	R	Susceptible	L4; S	LTFU
**7**	60	M	South	Positive	H	Susceptible	L4; LAM3	LTFU
**8**	1	M	Central	Unknown	H	Susceptible	L4; LAM4	LTFU
**9**	30	M	Central	Positive	H; R	Susceptible	L4; LAM3	Not Initiated
**10**	32	M	South	Unknown	H	Susceptible	L4; S	LTFU
**11**	35	F	Central	Positive	H; R; E; S	Susceptible	L2; Beijing	Cured
**12**	54	M	North	Positive	H; R	Susceptible	L2; Beijing	Deceased
**13**	23	M	South	Negative	H; R; S	Susceptible	L4; LAM3	Completed
**14**	33	M	South	Positive	H; R; E; S	Susceptible	L4; S	Not Initiated
**15**	39	M	South	Positive	R	Susceptible	L4; X3	Cured
**16**	40	M	North	Positive	H; R; E; S	Susceptible	L2; Beijing	Completed
**17**	42	M	South	Positive	H; R; E; S	Susceptible	L4; T1	Completed
**18**	32	F	North	Positive	H; R; E	Susceptible	L2; Beijing	Completed
**19**	28	M	South	Negative	H; R; E; S	Susceptible	L2; Beijing	Cured
**20**	63	M	South	Negative	H; R	Susceptible	L4; X3	Cured
**21**	72	F	South	Negative	R	Susceptible	L4; S	Completed
**22**	18	F	Central	Unknown	H; R	Susceptible	L4; LAM3	Defaulted
**23**	49	M	North	Negative	H; R; E; S	Susceptible	L1; EAI	Completed
**24**	52	M	North	Positive	H; R	Susceptible	L2; Beijing	Deceased
**25**	53	M	North	Unknown	H	Susceptible	L2; Beijing	LTFU
**26**	56	M	South	Unknown	H; R; E; S	Susceptible	L4; LAM3	Cured
**27**	30	F	North	Positive	H; R	Susceptible	L2; Beijing	Completed
**28**	19	F	South	Negative	R	Susceptible	L4; X3	LTFU
**29**	36	F	Central	Negative	R	Susceptible	L4; T1	Completed
**30**	40	M	Central	Positive	H; R; E; S	Susceptible	L1; EAI1_SOM	Completed
**31**	59	M	South	Negative	H; R; E; S	Susceptible	L1; EAI1_SOM	Cured
**32**	15	F	Central	Positive	H; R	Susceptible	L4; X2	Completed

pathogens

MDPI

**33**	34	F	South	Positive	H; R; E; S	Susceptible	Unknown; unknown	Deceased
**34**	40	F	Central	Positive	H; R; E; S	Susceptible	L1; EAI1_SOM	Failure
**35**	32	F	Central	Positive	H; R; E	Susceptible	L4; X2	Completed
**36**	32	M	South	Positive	H; R; E	Susceptible	L2; Beijing	LTFU
**37**	36	M	South	Negative	H	Susceptible	L4; T1	Deceased
**38**	13	M	North	Unknown	R	Susceptible	L4; T3	LTFU
**39**	33	M	South	Positive	R	Susceptible	L4; S	Not Initiated
**40**	35	M	Central	Positive	R	Susceptible	L4; S	Completed
**41**	21	F	South	Negative	R	Susceptible	L4; T2-T3	LTFU
**42**	33	F	South	Unknown	H; R; E; S	Susceptible	L4; X3	Completed
**43**	37	F	Central	Positive	H; R; E; S	Susceptible	L1; EAI1_SOM	Completed
**44**	8	F	North	Unknown	H	Susceptible	L4; S	LTFU
**45**	7	M	North	Negative	H; R; E	Susceptible	L4; LAM1	Completed
**46**	43	F	North	Positive	H; R; S	Susceptible	L4; X3	Completed
**47**	47	M	North	Positive	R	Susceptible	L4; LAM3	Completed
**48**	40	M	Central	Positive	R	Susceptible	L4; X3	LTFU
**49**	41	M	South	Unknown	H; R; E; S	Susceptible	L4; LAM11_ZWE	Completed
**50**	27	F	South	Positive	H; R; E; S	Susceptible	L4; LAM11_ZWE	Completed

LFTU: Lost to follow up; H: Isoniazid; R: Rifampicin; S: Streptomycin; E: Ethambutol; L1: Lineage 1; L2: Lineage 2; L4: Lineage 4
